# Supplementary material for: Multi-Enzymatic Cascades in the Synthesis of Modified Nucleosides: Comparison of the Thermophilic and Mesophilic Pathways
Source: Biomolecules. 2021 Apr 16;11(4):586. doi: 10.3390/biom11040586 (PMC8073115; doi:10.3390/biom11040586)
Supplement: Supplementary file 1 [file biomolecules-11-00586-s001.zip › biomolecules-1092493-supplementary (2).pdf]

Supplementary Materials

Multienzymatic cascades in the synthesis of modified nucleosides: comparison of the thermophilic and mesophilic pathways.

I.V. Fateev, M.A. Kostromina, Y.A.Abramchik, B.Z. Eletskaya, O.O. Mikheeva, D.D. Lukoshin, E.A. Zayats, M.Ya. Berzina, E.V. Dorofeeva, A.S. Paramonov, A.L. Kayushin, I.D. Konstantinova*, and R.S. Esipov

Content	Page number
**Figure S1.** Synthesis of 2-chloro-6-methoxypurine (**3**)	S-2
**Figure S2.** The ^{1}H NMR spectrum of compound **1**	S-2
**Figure S3.** The ^{1}H NMR spectrum of compound **2**	S-3
**Figure S4.** The ^{13}C NMR spectrum of compound **2**	S-4
**Figure S5.** The fragment of ^{1}H-^{15}N-HMBC NMR spectrum of nucleoside **2**	S-4
**Figure S6.** Synthesis of 2-fluoro-6-methoxypurine (**7**)	S-5
**Figure S7.** The ^{1}H NMR spectrum of compound **4**	S-5
**Figure S8.** The ^{1}H NMR spectrum of compound **5**	S-6
**Figure S9.** The ^{13}C NMR spectrum of compound **5**	S-7
**Figure S10.** The fragment of ^{1}H-^{15}N-HMBC NMR spectrum of nucleoside **5**	S-7
**Figure S11.** The ^{1}H NMR spectrum of compound **6**	S-8
**Figure S12.** The ^{13}C NMR spectrum of compound **6**	S-9
**Figure S13.** The fragment of ^{1}H-^{15}N-HMBC NMR spectrum of nucleoside **6**	S-9
**Figure S14.** The ^{1}H NMR spectrum of compound **8**	S-10
**Figure S15.** The fragment of ^{1}H-^{13}C-HSQC NMR spectrum of nucleoside **8**	S-11
**Figure S16.** The fragment of ^{1}H-^{15}N-HMBC NMR spectrum of nucleoside **8**	S-11
**Figure S17.** The ^{1}H NMR spectrum of compound **9**	S-12
**Figure S18.** The ^{13}C NMR spectrum of compound **9**	S-13
**Figure S19.** The fragment of ^{1}H-^{15}N-HMBC NMR spectrum of nucleoside **9**	S-13
**Figure S20.** The ^{1}H NMR spectrum of compound **10**	S-14
**Figure S21.** The ^{13}C NMR spectrum of compound **10**	S-15
**Figure S22.** The fragment of ^{1}H-^{15}N-HMBC NMR spectrum of nucleoside **10**	S-15
**Figure S23.** The ^{1}H NMR spectrum of compound **11**	S-16
**Figure S24.** The ^{13}C NMR spectrum of compound **11**	S-17
**Figure S25.** The fragment of ^{1}H-^{15}N-HMBC NMR spectrum of nucleoside **11**	S-17
**References**	S-18

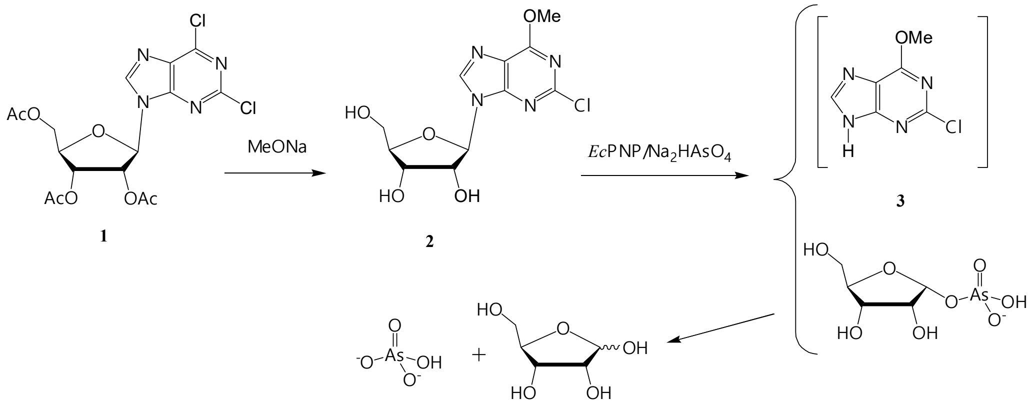

Figure S1. Synthesis of 2-chloro-6-methoxypurine (**3**)

9-(2,3,5-Tri-*O*-acetyl-β-D-ribofuranosyl)-2,6-dichloropurine (1)

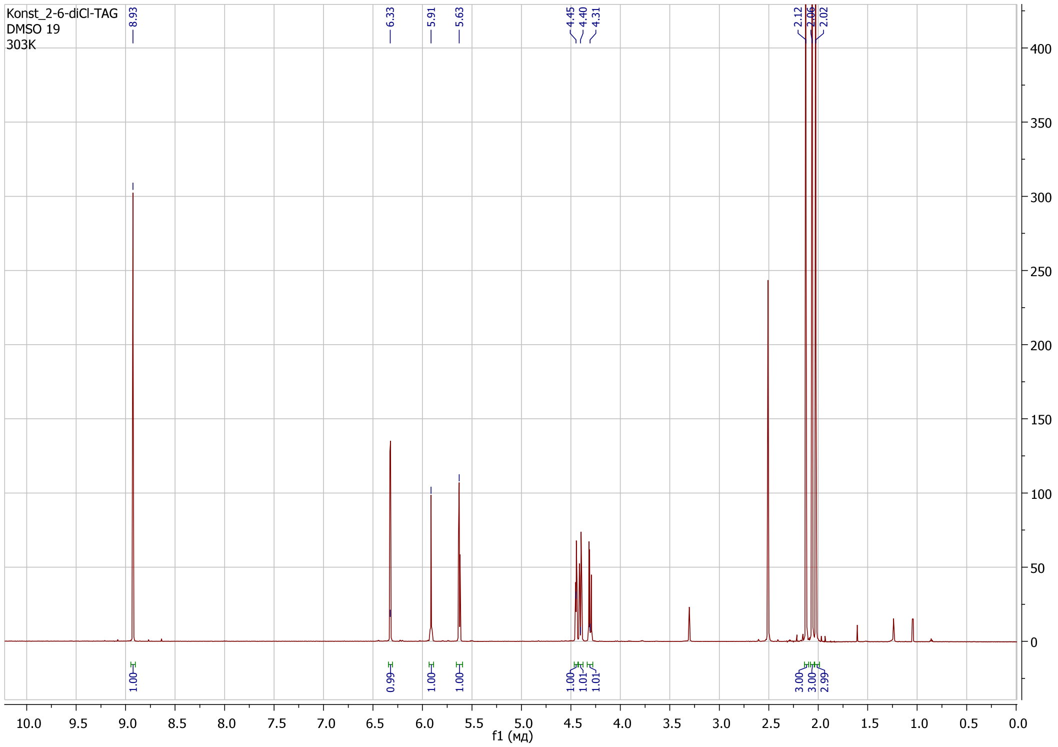

Figure S2. The ^{1}H NMR spectrum of compound **1**

^{1}H NMR (700 MHz, DMSO-d6, 30 °C): δ=8.93 (s, 1 H, H-8), 6.33 (d, $J_{H1,H2}$ = 5.0 Hz, 1 H, H-1′), 5.91 (t, J=5.5 Hz, 1 H, H-2′), 5.63 (t, J= 5.6 Hz, 1 H, H-3′), 4.45 (m, 1 H, H-4′), 4.40 (dd, J= 3.7, 12.12 Hz, 1 H, H-5′a), 4.31 (dd, J= 5.5, 12.1 Hz, 1 H, H-5′b), 2.12, 2.06, 2.02 (3 s, 9 H, 3 Me_{Ac}).[1, 2]

2-Chloro-6-methoxy-9-(β-D-ribofuranosyl)purine (2).

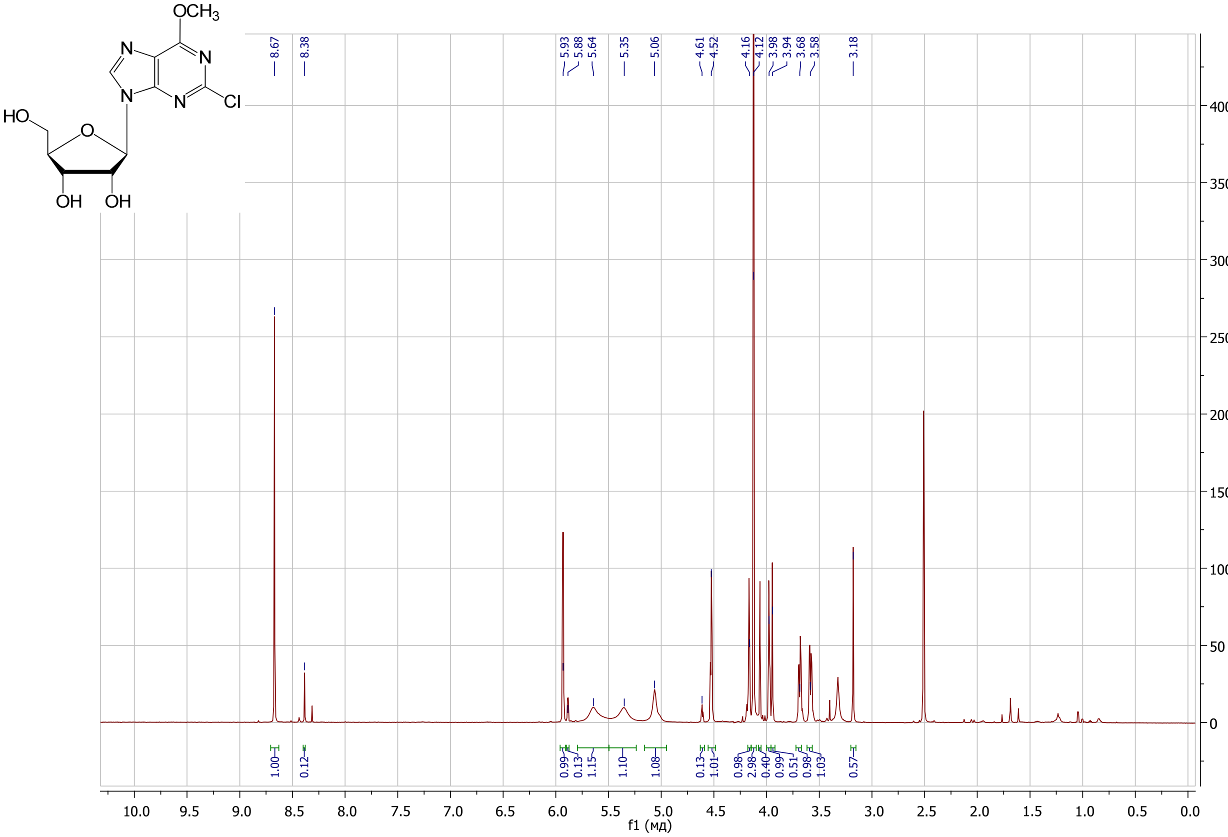

Figure S3. The ^{1}H NMR spectrum of compound **2**

^{1}H NMR (700 MHz, DMSO-d6, 30 °C): δ=8.67 (s, 1 H, H-8), 5.93 (d, J = 5.5 Hz, 1 H, H-1'), 5.64 and 5.35 (br. s, 2 H, OH-2' and OH-3'), 5.06 (m, 1 H, OH-5'), 4.52 (t, J = 5.1 Hz, 1 H, H-2'), 4.16 (t, J= 4.2 Hz, 1 H, H-3′), 4.12 (s, 3 H, OMe), 3.98 (m, 1 H, H-4′), 3.68 (dd, J= 4.0, 12.2 Hz, 1 H, H-5'a), 3.58 (dd, J= 3.7, 12.0 Hz, 1 H, H-5'b).
^{13}C NMR (176 MHz, DMSO-d6, 30 °C): δ=160.78 (C-6), 152.84 (C-4), 151.43 (C-2), 142.72 (C-8), 120.19 (C-5), 87.59 (C-1'), 85.63 (C-4'), 73.85 (C-2'), 70.05 (C-3'), 61.00 (C-5'), 54.89 (OCH_3).
^{15}N NMR (71 MHz, DMSO-d6, 30 °C): δ=241.3 (N-7), 172.47 (N-9).

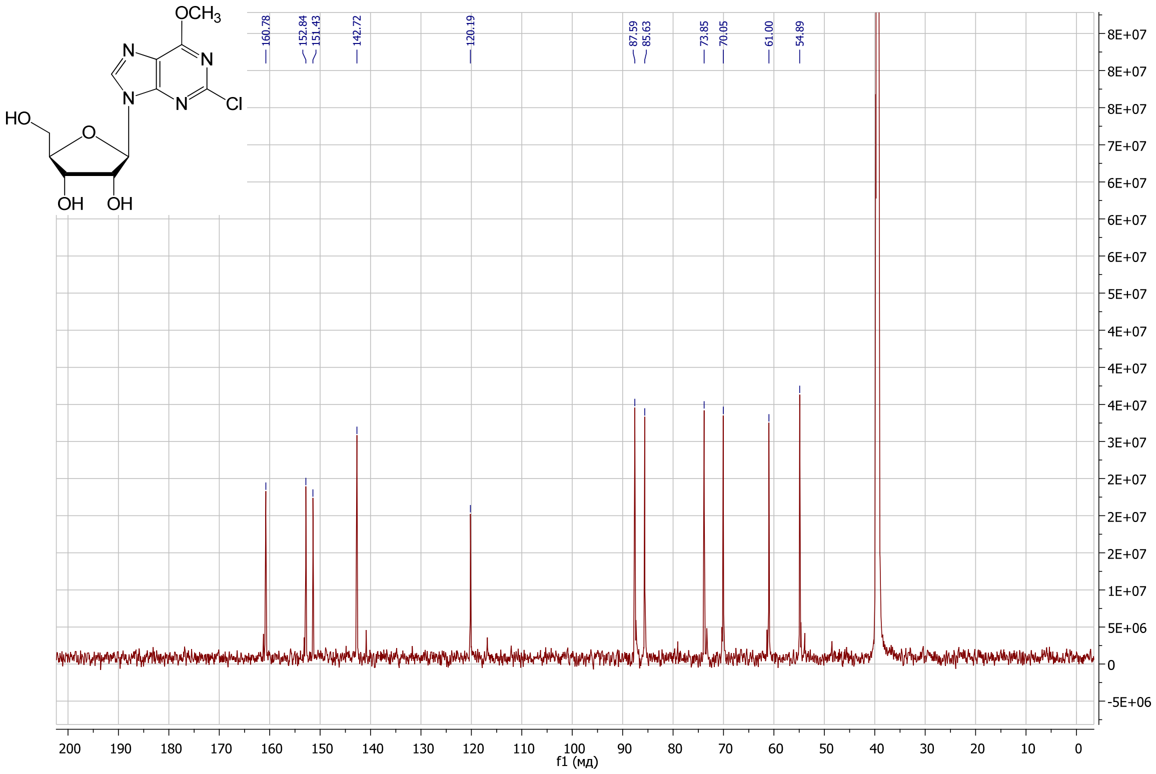

Figure S4. The ^{13}C NMR spectrum of compound **2**

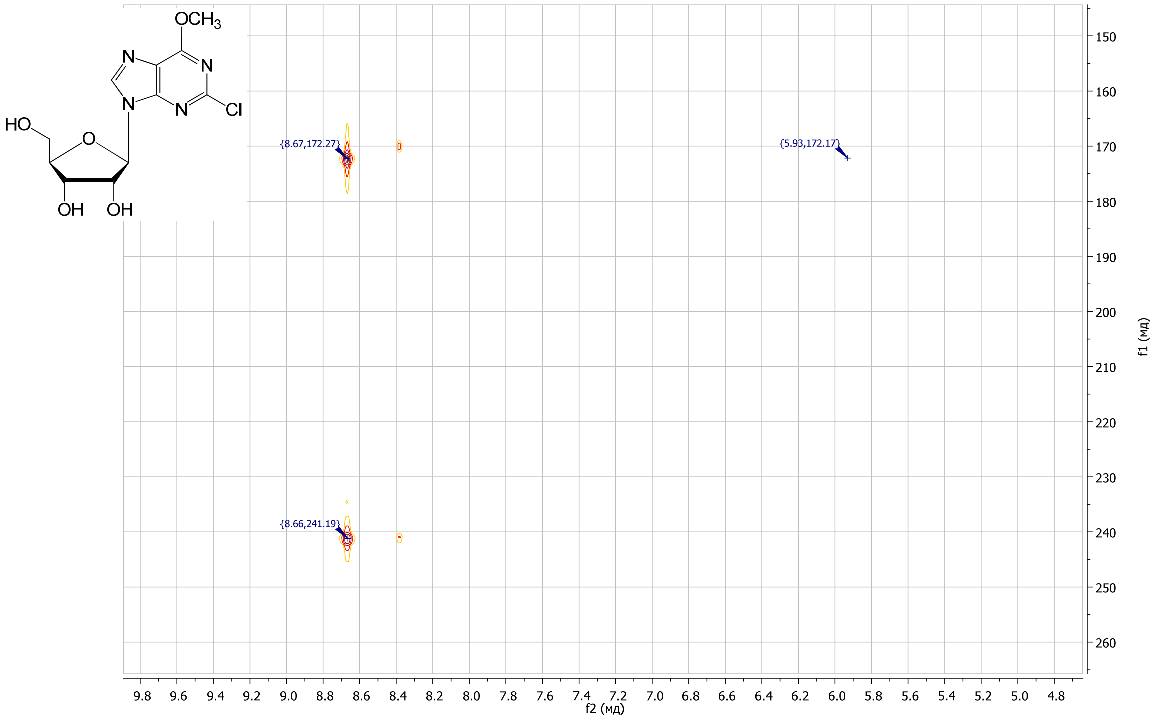

Figure S5. The fragment of ^{1}H-^{15}N-HMBC NMR spectrum of nucleoside **2**

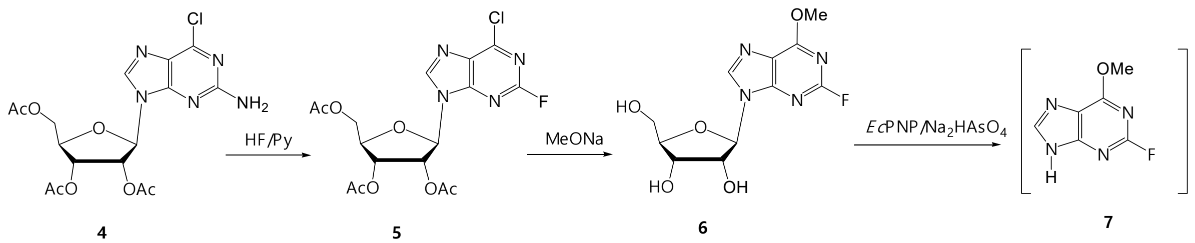

Figure S6. Synthesis of 2-fluoro-6-methoxypurine (**7**)

9-(2,3,5-Tri-*O*-acetyl-β-D-ribofuranosyl)-2-amino-6-chloropurine (4)

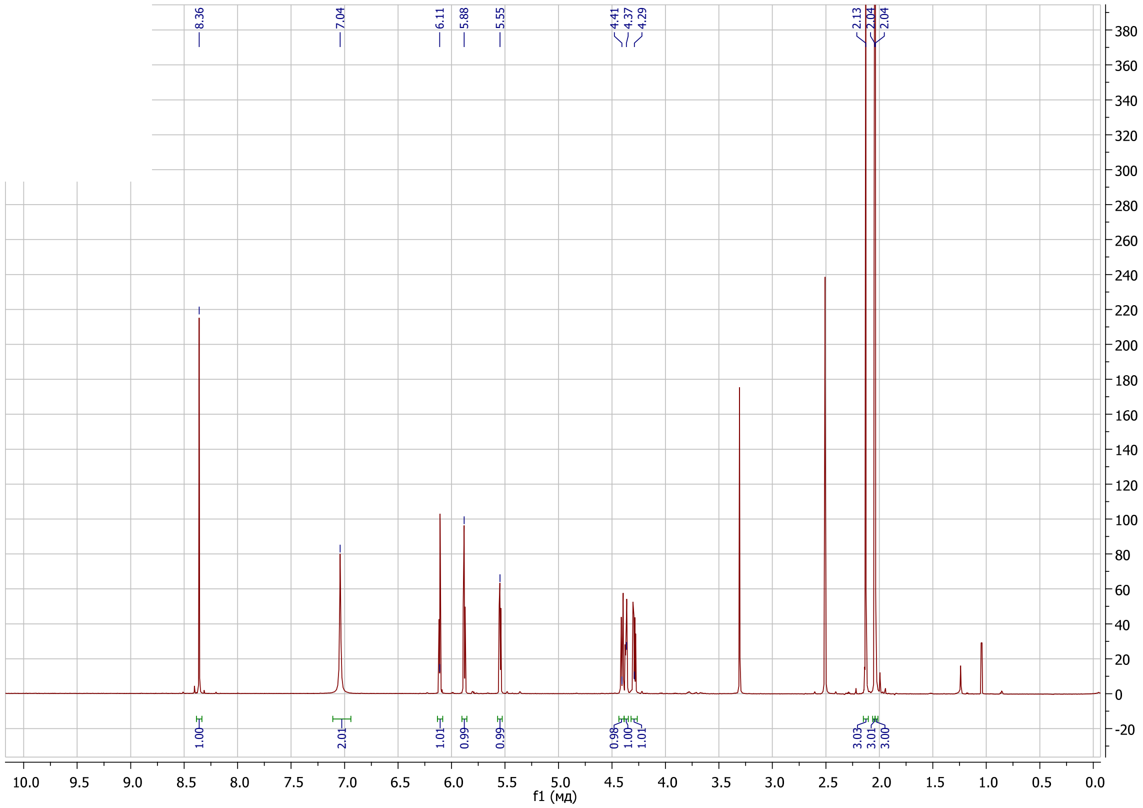

Figure S7. The ^{1}H NMR spectrum of compound **4**

^{1}H NMR (700 MHz, DMSO-d6, 30 °C): δ=8.36 (s, 1 H, H-8), 7.04 (s, 2 H, NH_2), 6.11 (d, $J_{H1,H2}$ = 5.8 Hz, 1 H, H-1′), 5.88 (t, *J* = 5.9 Hz, 1 H, H-2′), 5.55 (dd, *J* = 4.7, 5.7 Hz, 1 H, H-3′), 4.37 (m, 1 H, H-4′), 4.41 (dd, *J* = 4.1, 11.8 Hz, 1 H, H-5′a), 4.29 (dd, *J* = 5.8, 11.8 Hz, 1 H, H-5′b), 2.13, 2.043, 2.037 (3 s, 9 H, 3 Me_{Ac}).

9-(2,3,5-Tri-*O*-acetyl-β-D-ribofuranosyl)-2-fluoro-6-chloropurine (5)

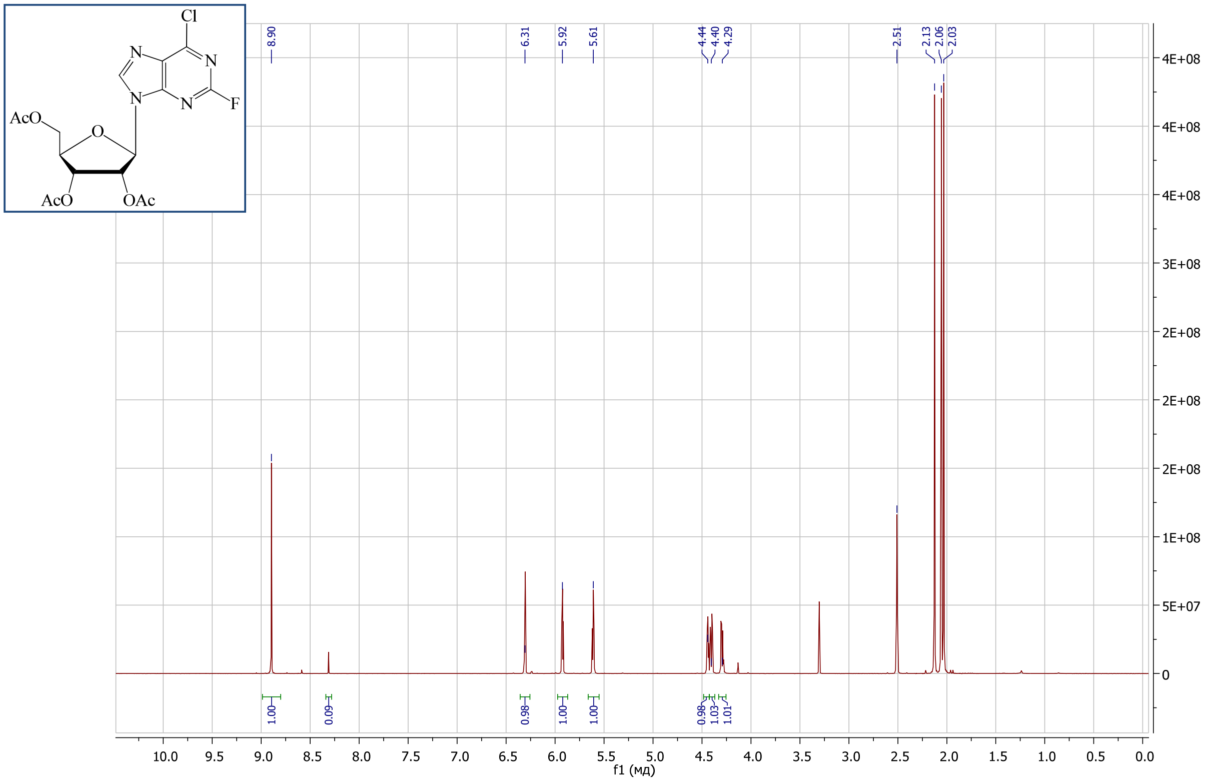

Figure S8. The ^{1}H NMR spectrum of compound **5**

^{1}H NMR (700 MHz, DMSO-d6, 30 °C): δ = 8.90 (s, 1 H, H-8), 6.31 (d, $J_{H1,H2}$ = 5.2 Hz, 1 H, H-1'), 5.92 (t, J = 5.5 Hz, 1 H, H-2'), 5.61 (t, J = 5.6 Hz, 1 H, H-3'), 4.44 (m, 1 H, H-4'), 4.40 (dd, J = 3.4, 12.2 Hz, 1 H, H-5'a), 4.29 (dd, J = 5.4, 12.2 Hz, 1 H, H-5'b), 2.13 (s, 3 H, $Me_{Ac3'}$), 2.06 (s, 3 H, $Me_{Ac2'}$), 2.03 (s, 3 H, $Me_{Ac5'}$).

^{13}C NMR (176 MHz, DMSO-d6, 30 °C): δ=169.88 (C=$O_{Ac5'}$), 169.24 (C=$O_{Ac3'}$), 169.09 (C=$O_{Ac2'}$), 156.01 (d, $J_{C2,F2}$ = 215.1 Hz, C-2), 153.12 (d, $J_{C4,F2}$ = 16.6 Hz, C-4), 150.95 (d, $J_{C6,F2}$ = 17.3 Hz, C-6), 146.79 (C-8), 130.65 (C-5), 86.09 (C-1'), 79.70 (C-4'), 72.01 (C-2'), 69.63 (C-3'), 62.51 (C-5'), 20.29 ($C_{MeAc5'}$), 20.22 ($C_{MeAc3'}$), 20.07 ($C_{MeAc2'}$).

^{15}N NMR (71 MHz, DMSO-d6, 30 °C): δ=243.03 (N-7), 169.79 (N-9).

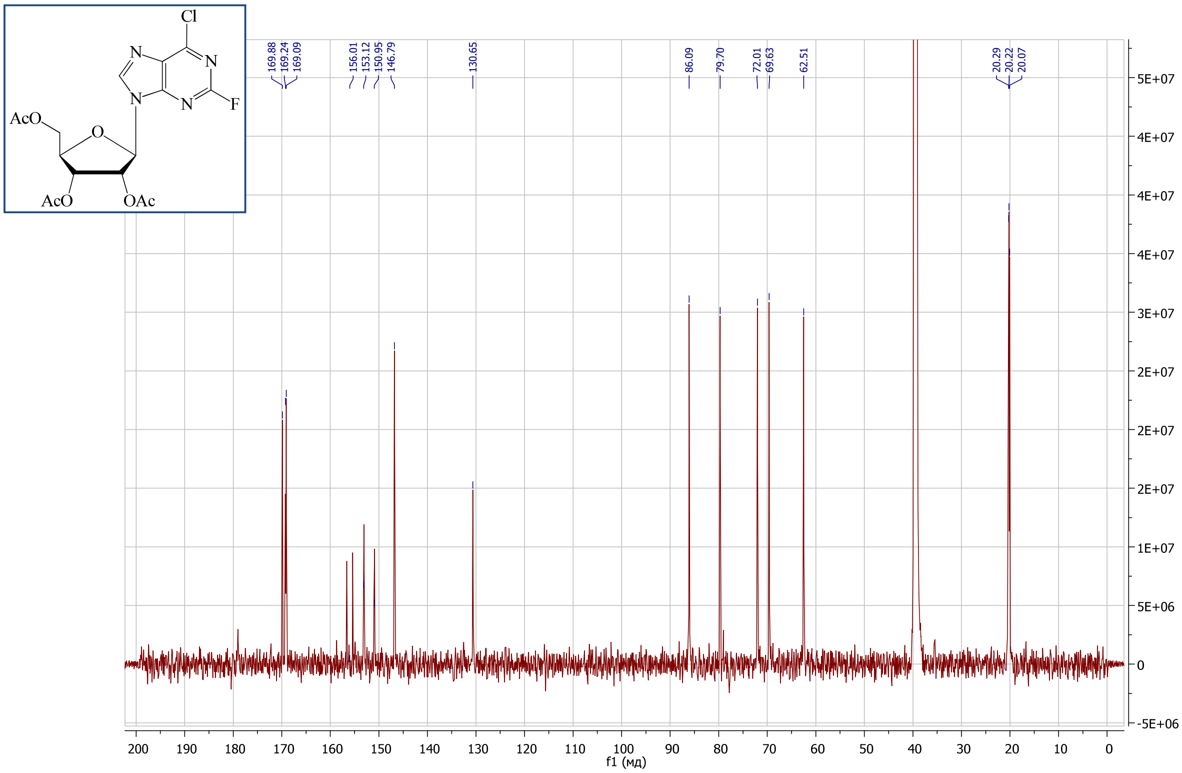

Figure S9. The ^{13}C NMR spectrum of compound **5**

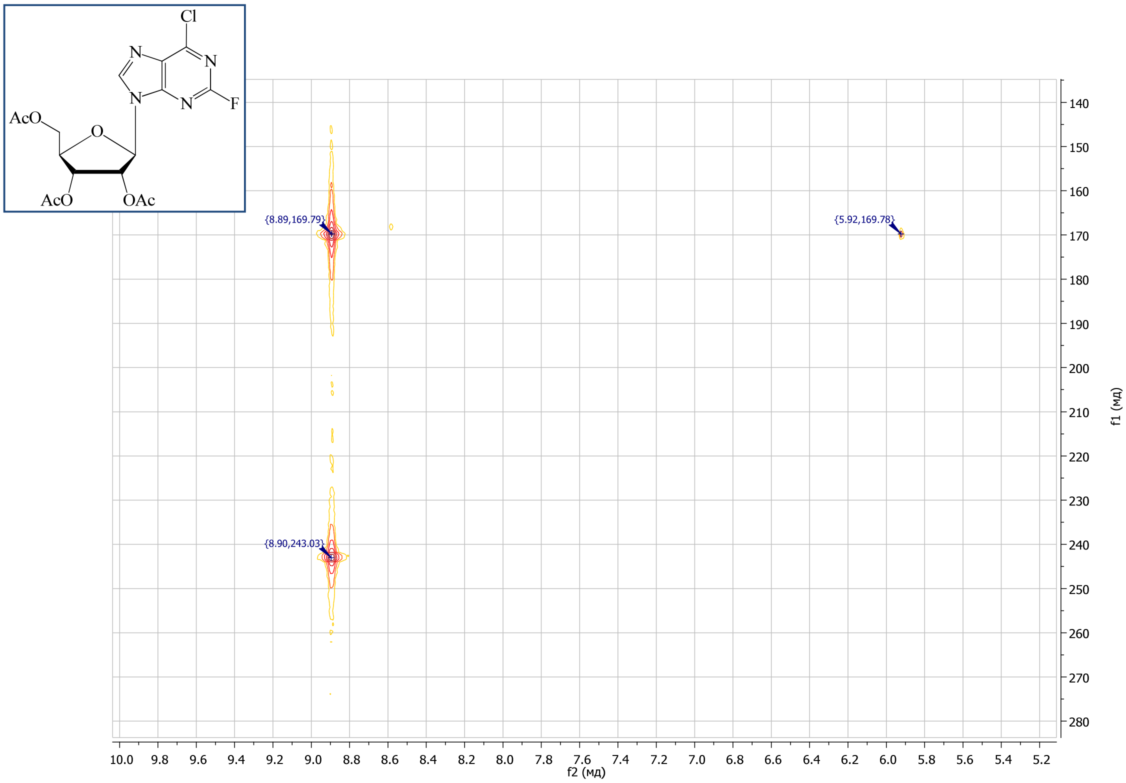

Figure S10. The fragment of ^{1}H-^{15}N-HMBC NMR spectrum of nucleoside **5**

2-Fluoro-6-methoxy-9-(β-D-ribofuranosyl)purine (6).

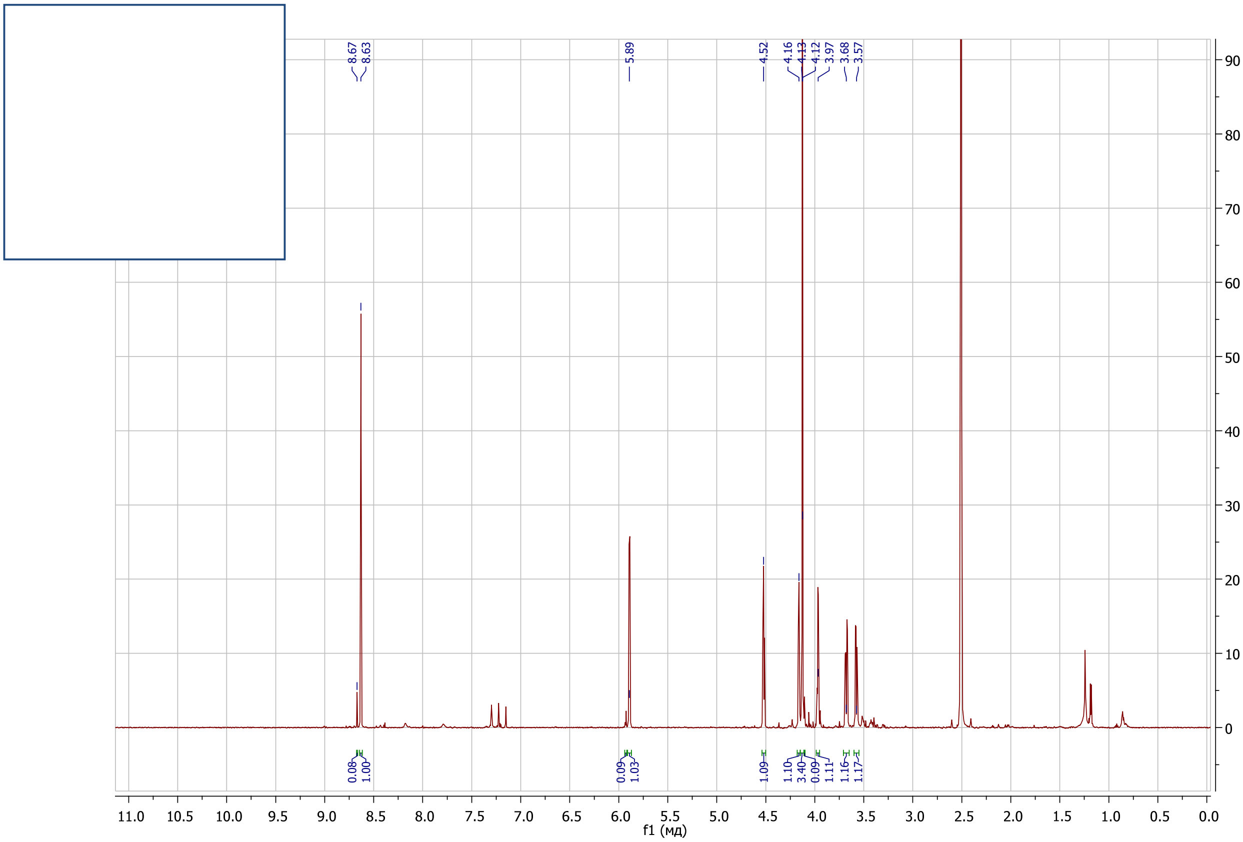

Figure S11. The ^{1}H NMR spectrum of compound **6**

^{1}H NMR (700 MHz, DMSO-d6, 30 °C): δ=8.63 (s, 1 H, H-8), 5.89 (d, $J_{H1,H2}$ = 5.5 Hz, 1 H, H-1’), 4.52 (t, J = 5.3 Hz, 1 H, H-2’), 4.16 (t, J= 4.3 Hz, 1 H, H-3′), 4.13 (s, 3 H, OMe), 3.97 (m, 1 H, H-4′), 3.68 (dd, J= 4.1, 12.0 Hz, 1 H, H-5’a), 3.57 (dd, J= 4.1, 12.0 Hz, 1 H, H-5’b).
^{13}C NMR (176 MHz, DMSO-d6, 30 °C):δ=162.16 (d, $J_{C6,F}$= 18.0, C-6), 156.97 (d, $J_{C2,F}$= 210.5, C-2), 152.89 (d, $J_{C4,F}$= 18.8, C-4), 142.53 (d, $J_{C8,F}$= 2.0, C-8), 119.35 (d, $J_{C5,F}$= 5.0, C-5), 87.57 (C-1′), 85.54 (C-4′), 73.73 (C-2′), 70.01 (C-3′), 60.98 (C-5′), 54.96 (OCH_3).

^{15}N NMR (71 MHz, DMSO-d6, 30 °C): δ=241.76 (N-7), 172.78 (N-9).

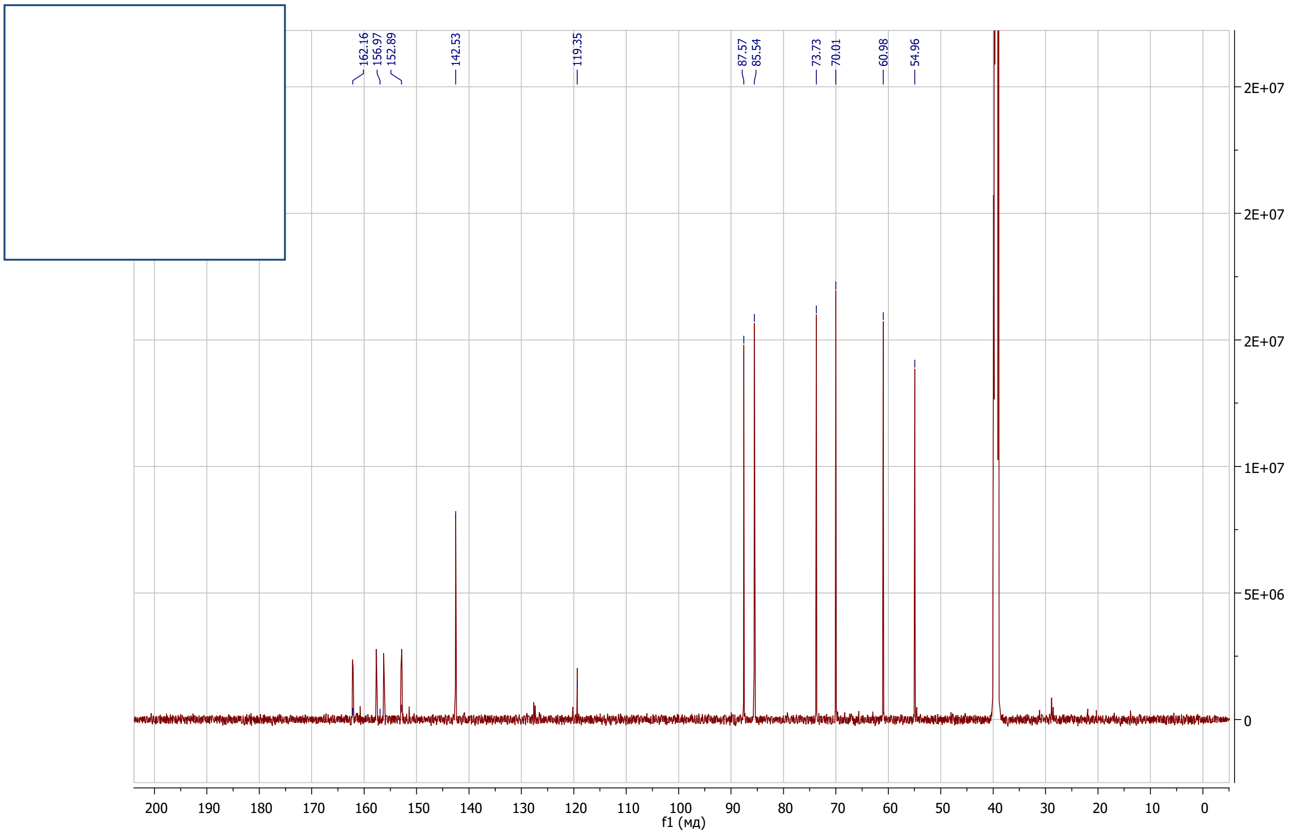

Figure S12. The ^{13}C NMR spectrum of compound **6**

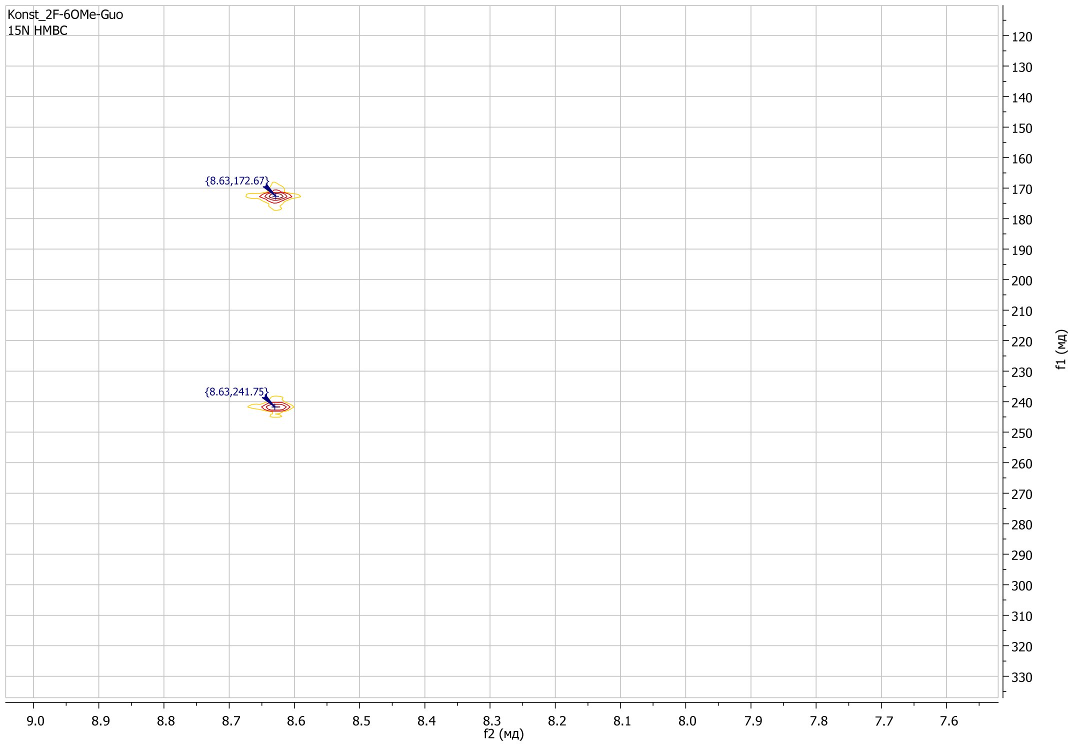

Figure S13. The fragment of ^{1}H-^{15}N-HMBC NMR spectrum of nucleoside **6**

5-Amino-3-(β-D-ribofuranosyl)triazolo[4,5-d]pyrimidin-7-one (8).

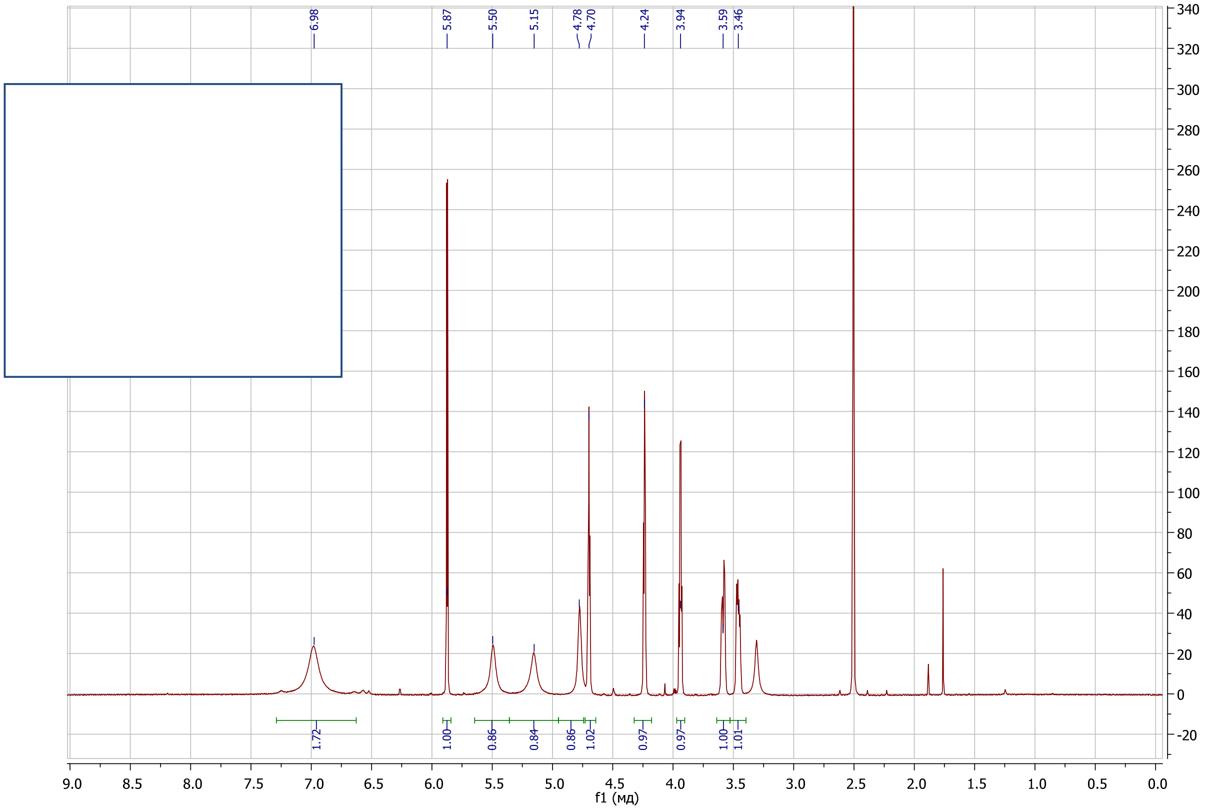

Figure S14. The ^{1}H NMR spectrum of compound **8**

^{1}H NMR (700 MHz, DMSO-d6, 30 °C): δ=6.98 (br.s, 2 H, NH_2), 5.87 (d, *J* = 4.8 Hz, 1 H, H-1′), 5.50 (s, 1 H, OH-2'), 5.15 (br. s, 1 H, OH-3'), 4.78 (m, 1 H, OH-5'), 4.70 (t, *J* = 4.9 Hz, 1 H, H-2′), 4.24 (t, *J*= 4.8 Hz, 1 H, H-3′), 3.94 (m, 1 H, H-4′), 3.59 (dd, *J*= 4.6, 11.9 Hz, 1 H, H-5′a), 3.46 (dd, *J*= 5.9, 11.7 Hz, 1 H, H-5′b).

^{13}C NMR (176 MHz, DMSO-d6, 30 °C): δ=151.73 (C-4), 87.75 (C-1′), 85.28 (C-4′), 72.47 (C-2′), 70.30 (C-3′), 61.62 (C-5′).

^{15}N NMR (71 MHz, DMSO-d6, 30 °C): δ=234.90, 156.95.

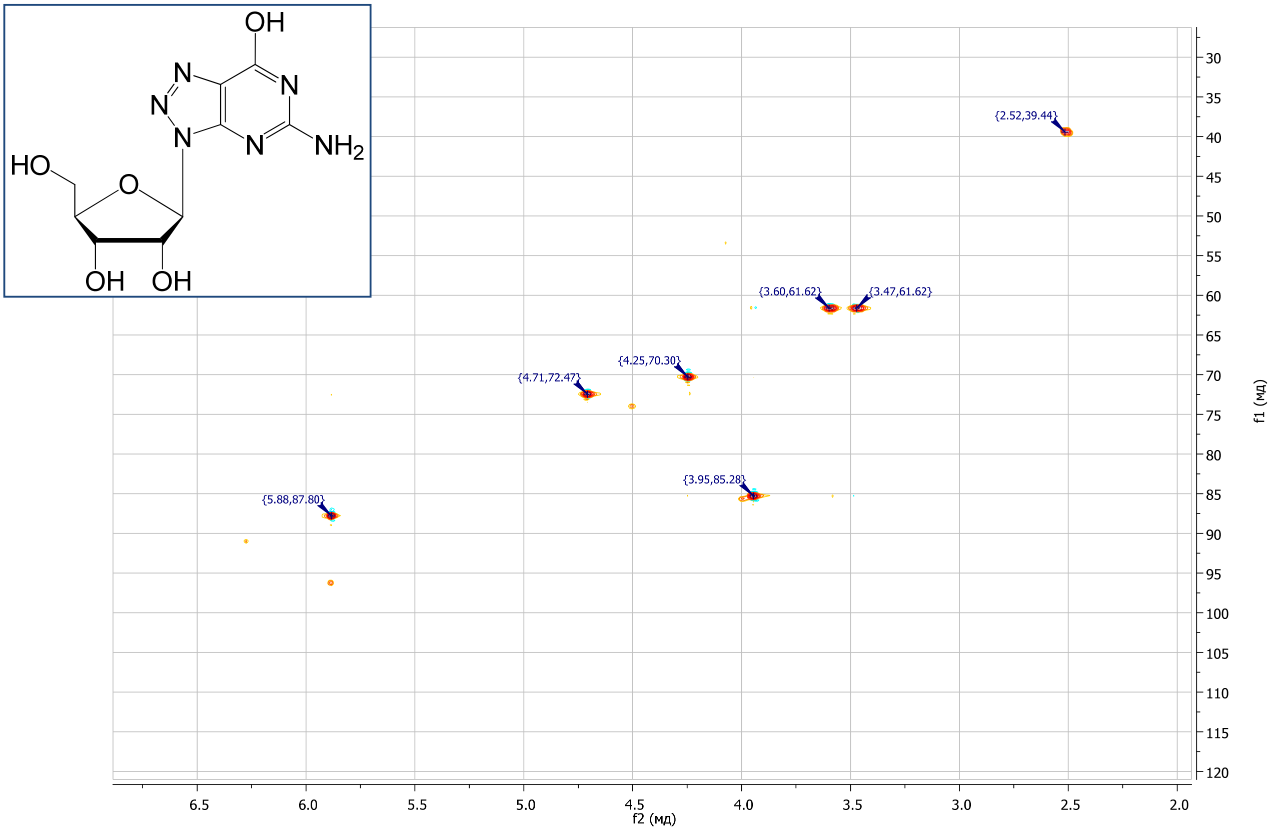

Figure S15. The fragment of ^{1}H-^{13}C-HSQC NMR spectrum of nucleoside **8**

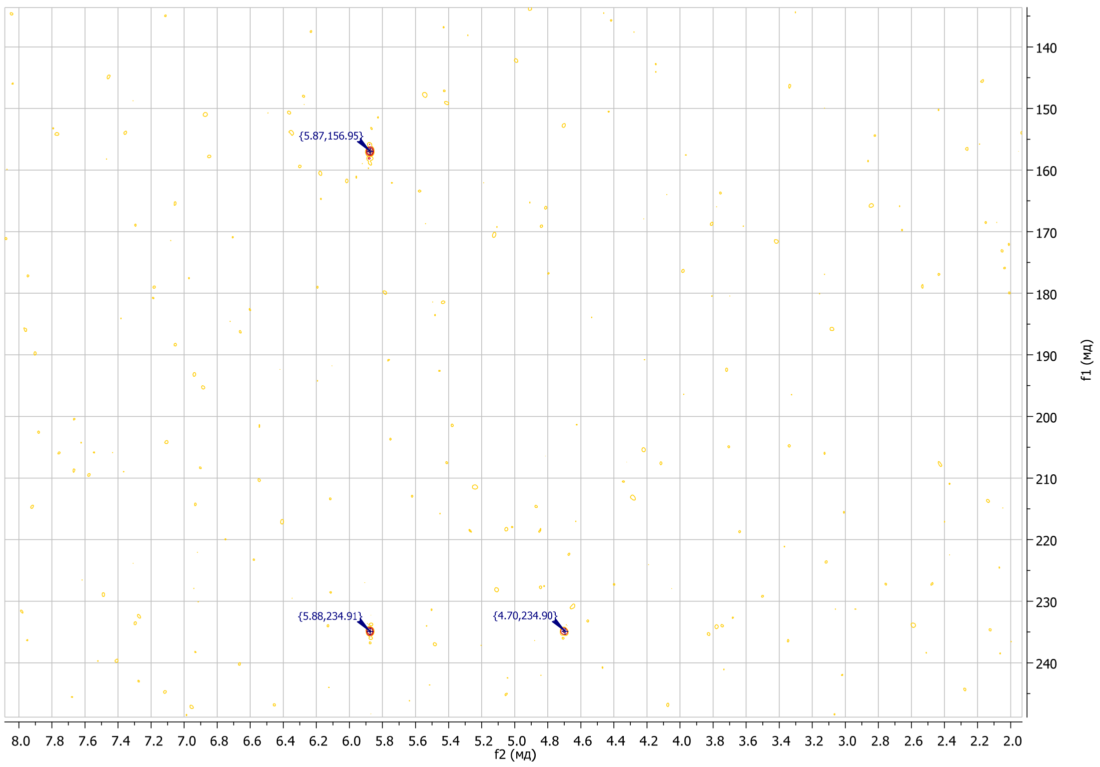

Figure S16. The fragment of ^{1}H-^{15}N-HMBC NMR spectrum of nucleoside **8**

1-(β-D-Ribofuranosyl)pyrazolo[3,4-d]pyrimidine-4-one (9).

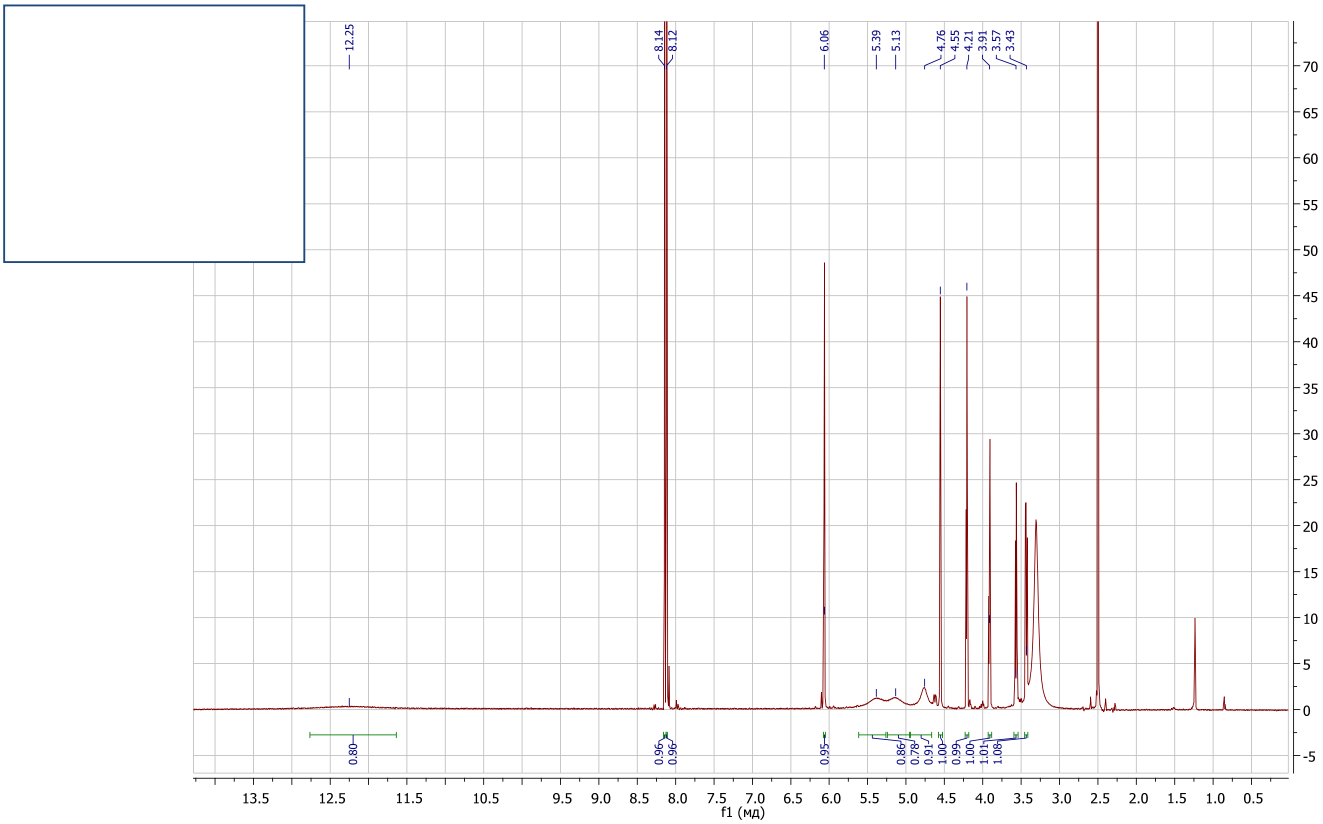

Figure S17. The ^{1}H NMR spectrum of compound **9**

^{1}H NMR (700 MHz, DMSO-d6): δ = 12.25 (br. s, 1 H, NH), 8.14 (s, 1 H, H-3), 8.12 (s, 1 H, H-6), 6.06 (d, *J* = 4.5 Hz, 1 H, H-1′), 5.39 (br. sign, 1H, OH-2’), 5.13 (br. sign, 1 H, OH-3’), 4.76 (br. sign, 1 H, OH-5’), 4.55 (dt, *J* = 4.6; < 0.5 Hz, 1 H, H-2′), 4.21 (t, *J* = 4.8 Hz, 1 H, H-3′), 3.91 (m, 1 H, H-4′), 3.58 (dd, *J*= 4.6, 11.7 Hz, 1 H, H-5′a), 3.43 (dd, *J* = 6.0, 11. 8 Hz, 1 H, H-5′b).

^{13}C NMR (176 MHz, DMSO-d6): δ = 157.45 (C-4), 153.98 (C-7a), 148.86 (C-6), 135.37 (C-3), 106.23 (C-4a), 88.38 (C-1′), 85.25 (C-4′), 73.31 (C-2′), 70.81 (C-3′), 62.27 (C-5′).

^{15}N NMR (71 MHz, DMSO-d6): δ = 302.20 (N-2), 210.47 (N-7), 204.80 (N-1), 173.67 (N-5).

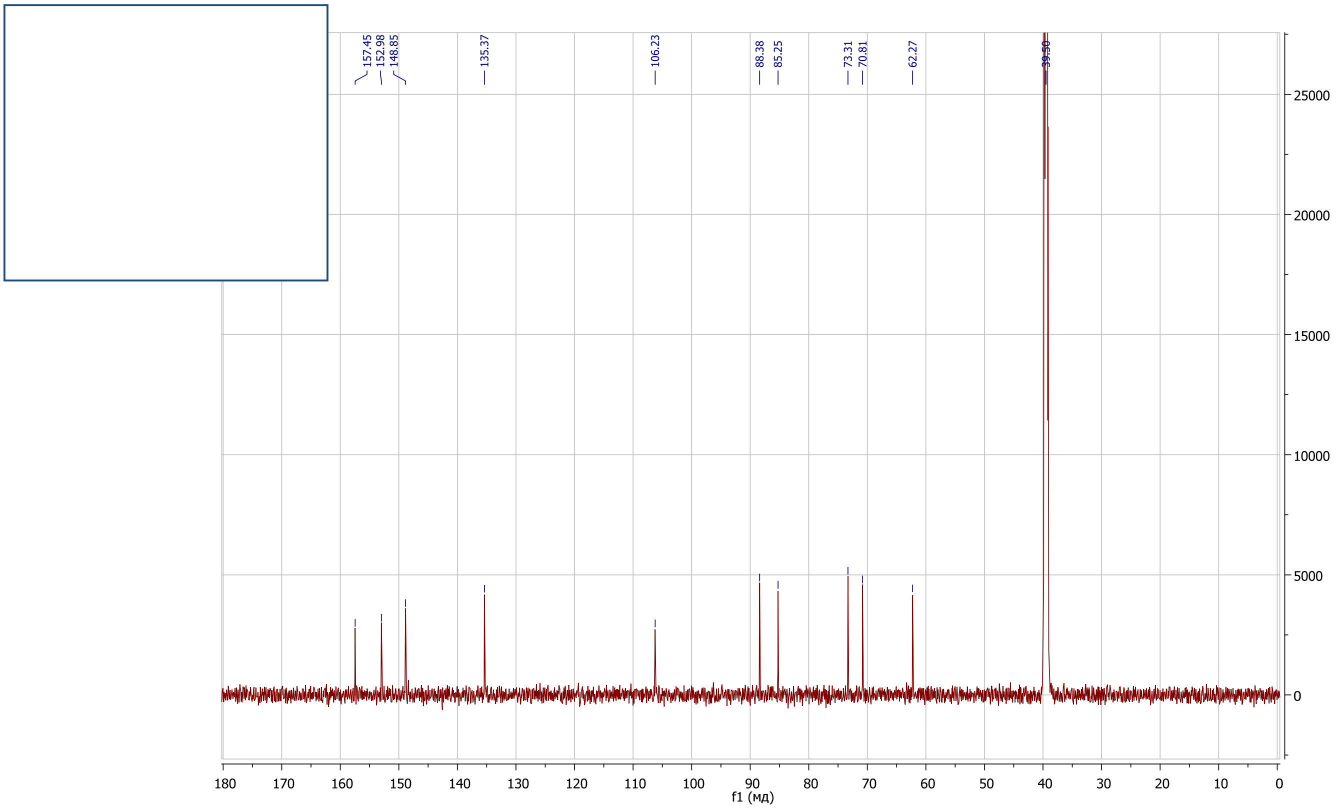

Figure S18. The ^{13}C NMR spectrum of compound **9**

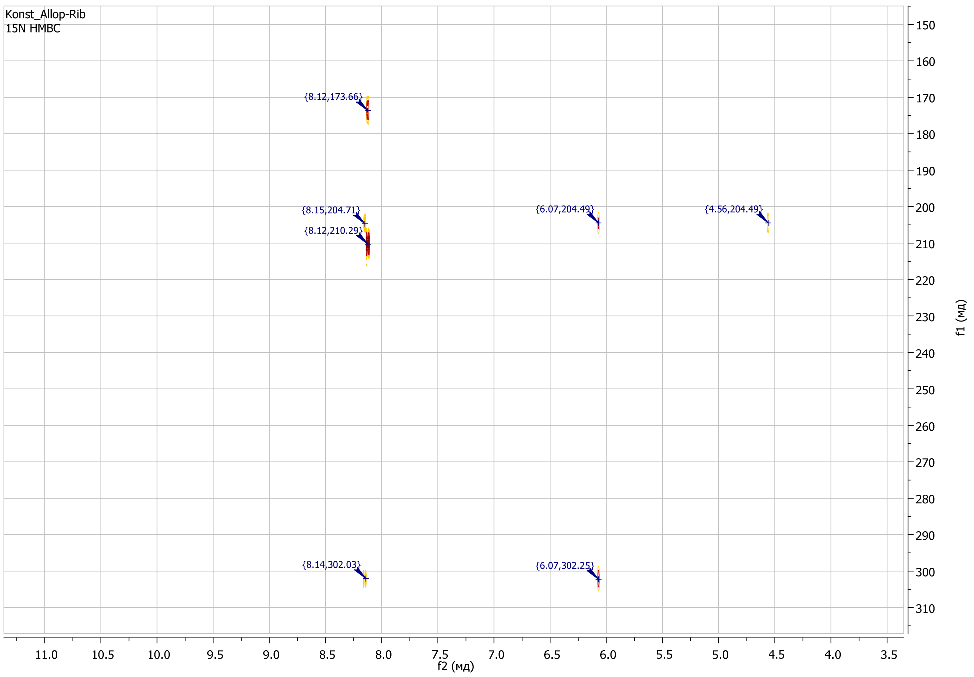

Figure S19. The fragment of ^{1}H-^{15}N-HMBC NMR spectrum of nucleoside **9**

9-(β-D-Arabinofuranosyl)-2-chloro-6-methoxypurine (10).

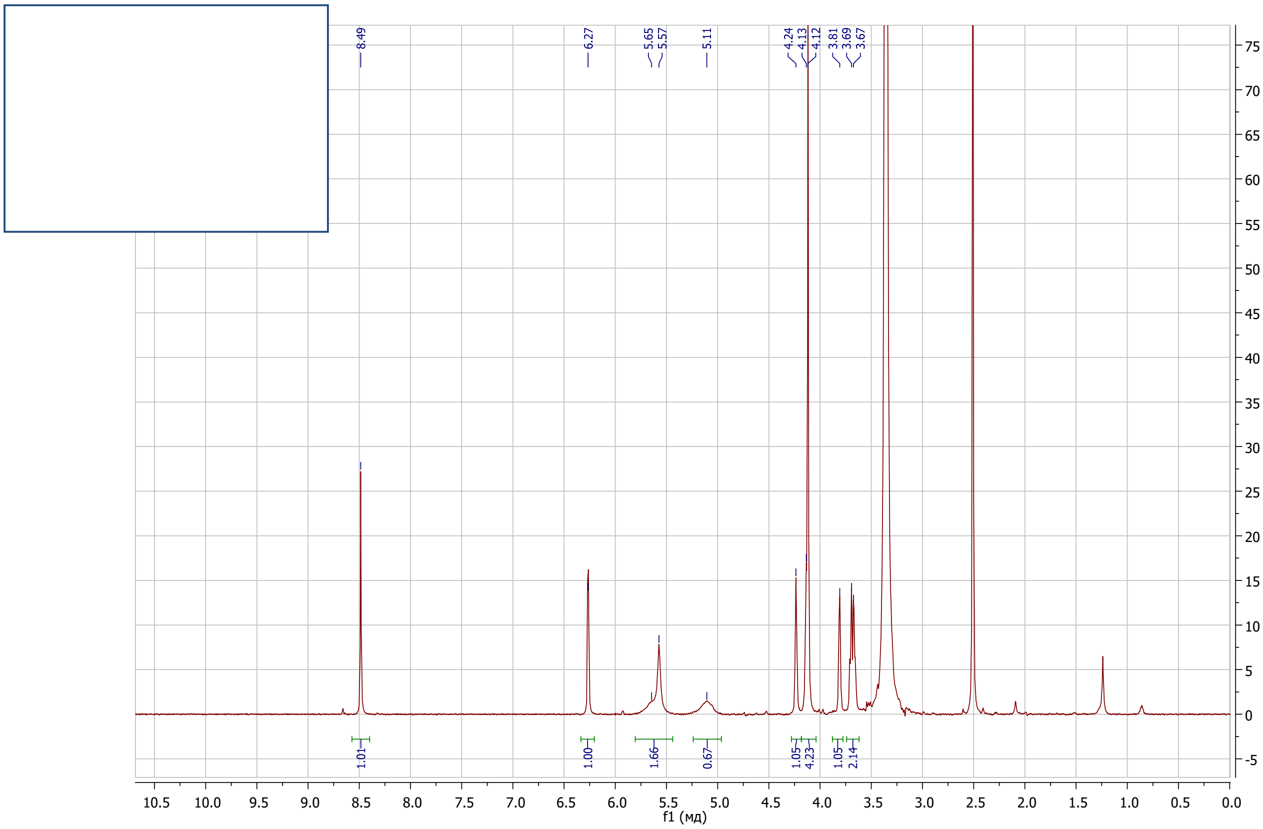

Figure S20. The ^{1}H NMR spectrum of compound **10**

^{1}H NMR (700 MHz, DMSO-d_6, 30 °C): δ=8.49, (s, 1 H, H-8), 6.27 (d, *J* = 4.8 Hz, 1 H, H-1′), 5.65 (br. s., 1 H, OH-2′), 5.57 (br. s, 1 H, OH-3′), 5.11 (br. s, 1 H, OH-5′), 4.24 (m, 1 H, H-2′), 4.13 (m, 1 H, H-3′), 4.12 (s, 3 H, O-CH_3), 3.81(m, 1 H, H-4′), 3.69 (m, 1 H, H-5′a), 3.67 (m, 1 H, H-5′b).

^{13}C NMR (176 MHz, DMSO-d_6, 30 °C): δ= 160.59 (C-6), 152.86 (C-4), 151.19 (C-2), 143.53 (C-8), 119.44 (C-5), 84.08 (C-4′), 84.06 (C-1′), 75.52 (C-2′), 74.19 (C-3′), 60.40 (C-5′), 54.83 (O-CH_3).

^{15}N ЯМР (71 МГц, ДМСО-d_6, 30 °C): δ=239.34 (N-7), 170.3 (N-9).

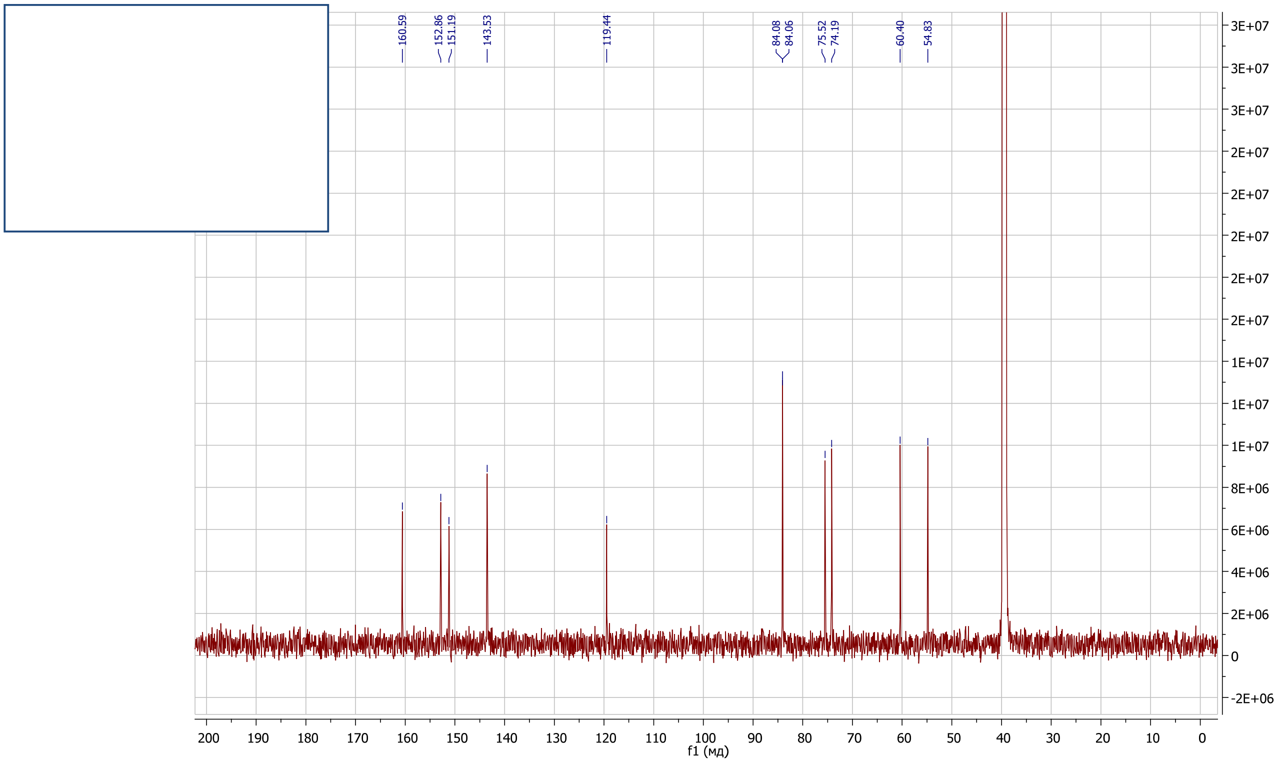

Figure S21. The ^{13}C NMR spectrum of compound **10**

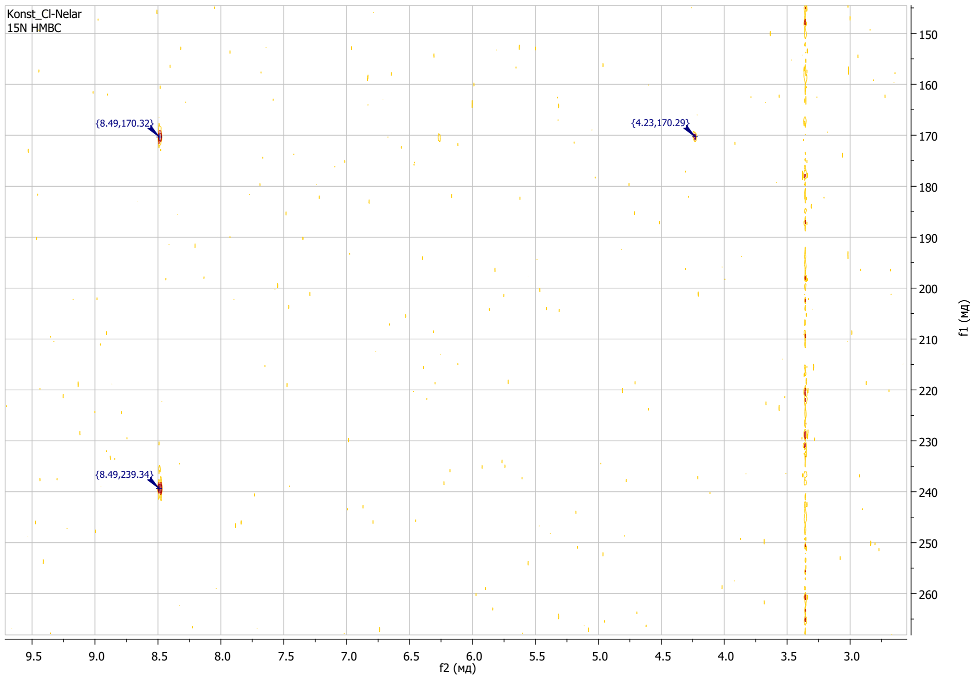

Figure S22. The fragment of ^{1}H-^{15}N-HMBC NMR spectrum of nucleoside **10**

9-(β-D-Arabinofuranosyl)-2-fluoro-6-methoxypurine (11).

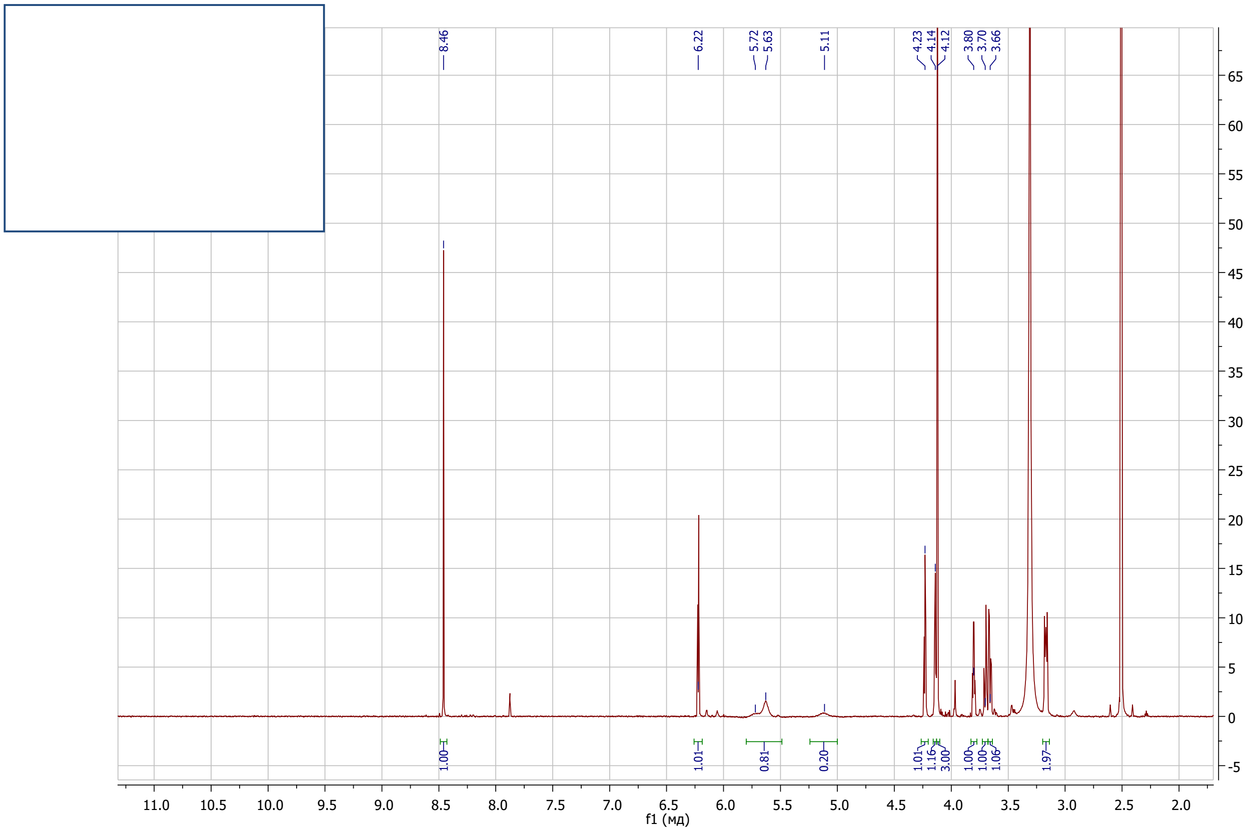

Figure S23. The ^{1}H NMR spectrum of compound **11**

^{1}H NMR (700 MHz, DMSO-d_6, 30 °C),: δ=8.46, (s, 1 H, H-8), 6.22 (d, *J* = 5.4 Hz, 1 H, H-1′), 5.72 (br. s., 1 H, OH-2′), 5.63 (br. s, 1 H, OH-3′), 5.11 (br. s, 1 H, OH-5′), 4.23 (t, *J* = 5.2 Hz, 1 H, H-2′), 4.14 (t, *J* = 5.4 Hz, 1 H, H-3′), 4.12 (s, 3 H, O-CH_3), 3.80(m, 1 H, H-4′), 3.70 (dd, *J* = 3.7, 12.0 Hz, 1 H, H-5′a), 3.66 (dd, *J* = 4.8, 12.0 Hz, 1 H, H-5′b).

^{13}C NMR (176 MHz, DMSO-d_6, 30 °C): δ= 161.98 (d, $J_{C6,F}$ = 17.0 Hz, C-6), 156.98 (d, $J_{C2,F}$ = 210.8 Hz, C-2), 153.00 (d, $J_{C4,F}$ = 18.8 Hz, C-4), 143.38 (C-8), 118.64 (d, $J_{C5,F}$ = 4.5 Hz, C-5), 84.09 (C-4′), 84.05 (C-1′), 75.59 (C-2′), 74.18 (C-3′), 60.44 (C-5′), 54.97 (O-CH_3).

^{15}N ЯМР (71 МГц, ДМСО-d_6, 30 °C): δ=239.9 (N-7), 200.11 (N-3), 170.71 (N-9).

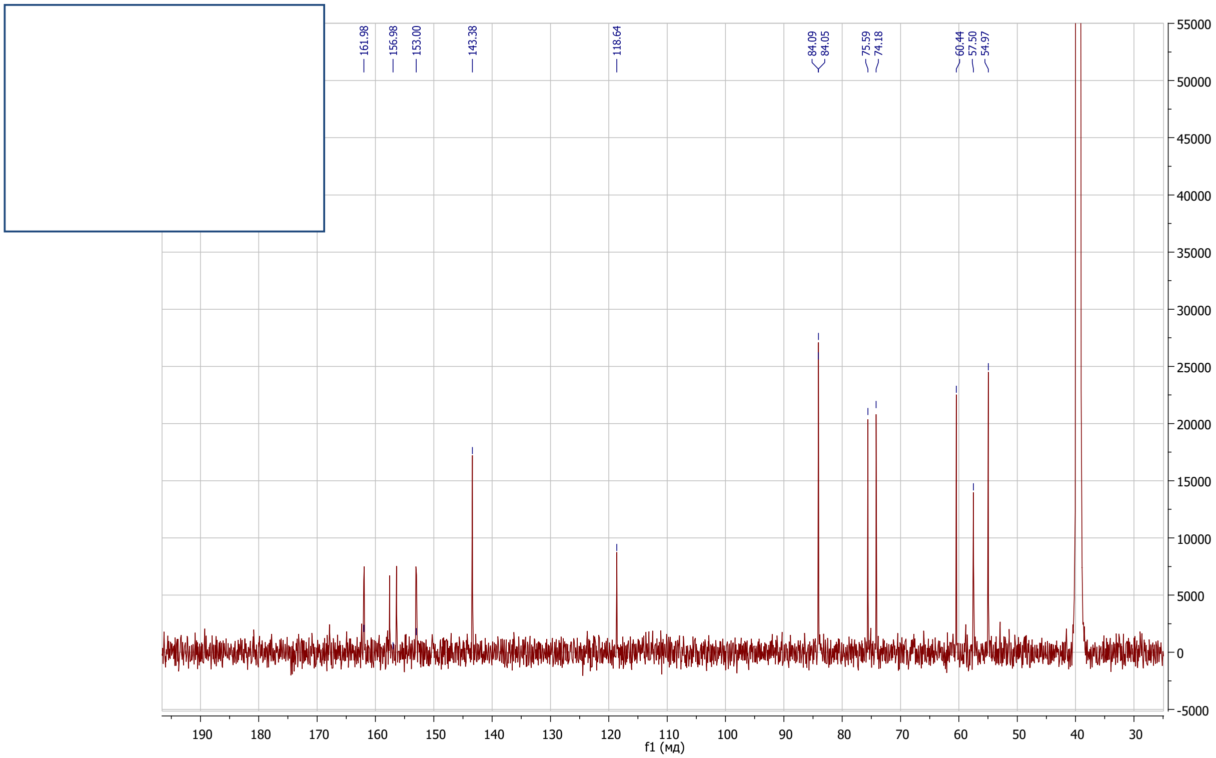

Figure S24. The ^{13}C NMR spectrum of compound **11**

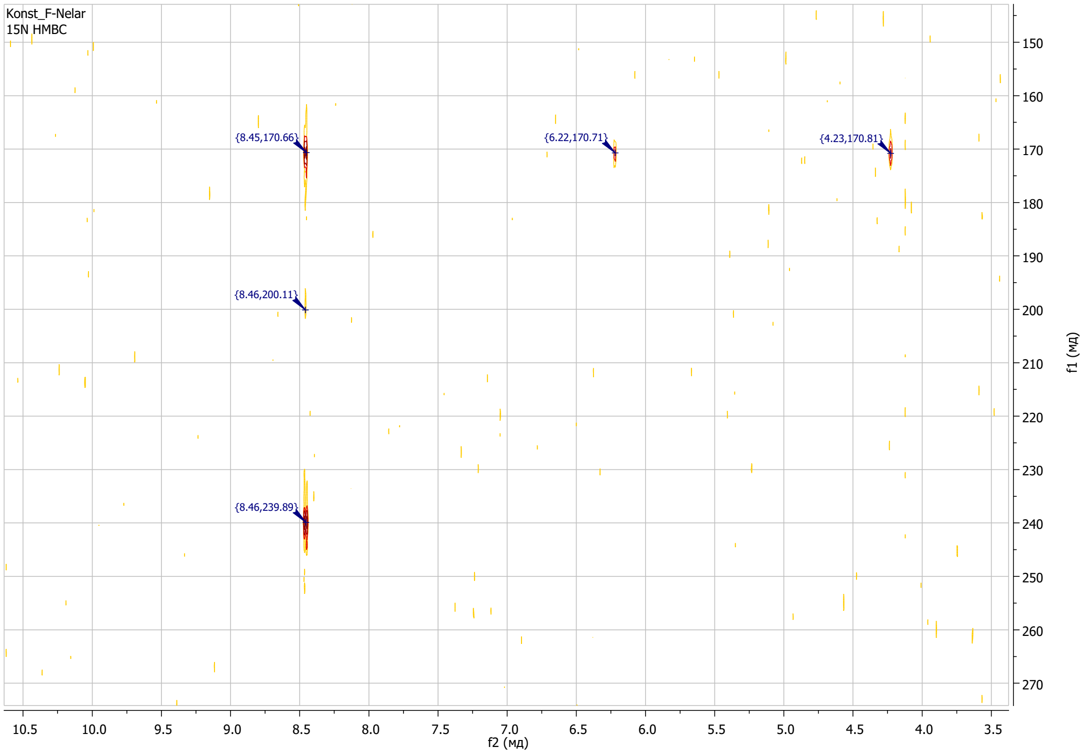

Figure S25. The fragment of ^{1}H-^{15}N-HMBC NMR spectrum of nucleoside **11**

References

1. Robins, M. J.; Uznański, B., Nucleic acid related compounds. 33. Conversions of adenosine and guanosine to 2, 6-dichloro, 2-amino-6-chloro, and derived purine nucleosides. *Canadian Journal of Chemistry* **1981,** *59* (17), 2601-2607.

2. Robins, M. J.; Zou, R.; Hansske, F.; Madej, D.; Tyrrell, D. L. J., Synthesis, Transformation Chemistry, and Biological Activity of Guanine Nucleosides and Analoges. *Nucleosides & Nucleotides* **1989,** *8* (5-6), 725-741.
